# Local Delivery Is Critical for Monocyte Chemotactic Protein-1 Mediated Site-Specific Murine Aneurysm Healing

**DOI:** 10.3389/fneur.2018.00158

**Published:** 2018-03-19

**Authors:** Siham Hourani, Kartik Motwani, Daisuke Wajima, Hanain Fazal, Chad H. Jones, Sylvain Doré, Koji Hosaka, Brian L. Hoh

**Affiliations:** ^1^Department of Neurosurgery, University of Florida, Gainesville, FL, United States; ^2^Department of Anesthesiology, University of Florida, Gainesville, FL, United States

**Keywords:** inflammation, monocyte chemotactic protein-1, CCR2, endovascular, coil, aneurysm, healing

## Abstract

**Background:**

Local delivery of monocyte chemotactic protein-1 (MCP-1/CCL2) *via* our drug-eluting coil has been shown to promote intrasaccular aneurysm healing *via* an inflammatory pathway.

**Objective:**

In this study, we validate the importance of local MCP-1 in murine aneurysm healing. Whether systemic, rather than local, delivery of MCP-1 can direct site-specific aneurysm healing has significant translational implications. If systemic MCP-1 is effective, then MCP-1 could be administered as a pill rather than by endovascular procedure. Furthermore, we confirm that MCP-1 is the primary effector in our MCP-1 eluting coil-mediated murine aneurysm healing model.

**Methods:**

We compare aneurysm healing with repeated intraperitoneal MCP-1 versus vehicle injection, in animals with control poly(lactic-co-glycolic) acid (PLGA)-coated coils. We demonstrate elimination of the MCP-1-associated tissue-healing response by knockout of MCP-1 or CCR2 (MCP-1 receptor) and by selectively inhibiting MCP-1 or CCR2. Using immunofluorescent probing, we explore the cell populations found in healed aneurysm tissue following each intervention.

**Results:**

Systemically administered MCP-1 with PLGA coil control does not produce comparable aneurysm healing, as seen with MCP-1 eluting coils. MCP-1-directed aneurysm healing is eliminated by selective inhibition of MCP-1 or CCR2 and in MCP-1-deficient or CCR2-deficient mice. No difference was detected in M2 macrophage and myofibroblast/smooth muscle cell staining with systemic MCP-1 versus vehicle in aneurysm wall, but a significant increase in these cell types was observed with MCP-1 eluting coil implant and attenuated by MCP-1/CCR2 blockade or deficiency.

**Conclusion:**

We show that systemic MCP-1 concurrent with PLGA-coated platinum coil implant is not sufficient to produce site-specific aneurysm healing. MCP-1 is a critical, not merely complementary, actor in the aneurysm healing pathway.

## Introduction

Intracranial aneurysms are estimated to occur in 1 in every 20–50 individuals, with about 50% mortality within 30 days if the aneurysm ruptures (1). Endovascular intrasaccular coiling is used widely in the management of unruptured and ruptured intracranial aneurysms. However, recanalization remains a significant concern (2). While current endovascular treatments aim to prevent unruptured aneurysms from rupturing, or a ruptured aneurysm from re-bleeding, they still carry inherent recurrence risk.

Complete and durable endovascular coil embolization requires complete wound healing to occur within the aneurysm sac. With the best possible results with platinum coil embolization, there is about 20–30% coil packing density of the intrasaccular space (3). The remaining 70–80% of intrasaccular space is filled with thrombus. As thrombus dissipates, there is “a race against time” for wound healing to occur. If wound healing is completed before the thrombus completely dissipates, then there is likely chance for durable cure. If complete wound healing is not achieved, any space between the coil and intraluminal tissue risk aneurysm recurrence.

Analyses of complete and incomplete aneurysmal repair of human intracranial aneurysms suggest successful healing contains neutrophils, vascular smooth muscle cells (vSMCs), myofibroblasts, endothelial cells, and macrophages (4–6). Monocyte chemotactic protein-1 (MCP-1, also known as CCL2) is known to mediate the infiltration of T-lymphocytes, monocytes, and macrophages, and notably involves acute inflammatory responses of tissue healing (7–9).

In this study, we ask whether systemic MCP-1, as opposed to local delivery, can produce biologically significant aneurysm tissue-healing. If systemic MCP-1 can direct site-specific aneurysm healing, then MCP-1 could be administered *via* a systemic route rather than coated on a device that requires an endovascular procedure. Previous studies on MCP-1 in other models have shown systemic MCP-1 can direct site-specific neutrophil infiltration, mesenchymal stem cell recruitment, and inflammatory and nociceptive mediators in various organs, such as in lung, heart, kidney, and post-surgical wound healing (10–13).

We have previously shown local delivery of MCP-1 to the aneurysm promotes inflammatory tissue ingrowth composed of macrophages and vSMCs (14). While we show intrasaccular MCP-1 delivery promotes aneurysm healing, we need to validate this finding to determine that MCP-1 is the critical component in the pathway. Otherwise, the observed aneurysm tissue healing may be due to another yet unidentified aspect of the MCP-1-polymer-coil construct in our experimental model. Or perhaps, MCP-1 may have a complementary, not critical role in aneurysm healing.

In contrast to surgically implanted local MCP-1-eluting coil, we measure the ability of systemic injection of MCP-1 to direct site-specific tissue-healing within the aneurysm. In addition, we validate that MCP-1 is in fact a critical cytokine in the aneurysm healing cascade by evaluating tissue-healing response with knockout (KO) or blockade of either MCP-1 or its receptor CCR2.

## Materials and Methods

All animal experiments were performed in accordance with approved protocol #201604771 from the University of Florida Institutional Animal Care and Use Committee and comply with Animal Research: Reporting of *In Vivo* Experiments guidelines.

Detailed materials and methods are included in Supplemental Material.

## Results

### Effect of Systemic Administration of MCP-1 on Aneurysm Healing

**Figure 1:**
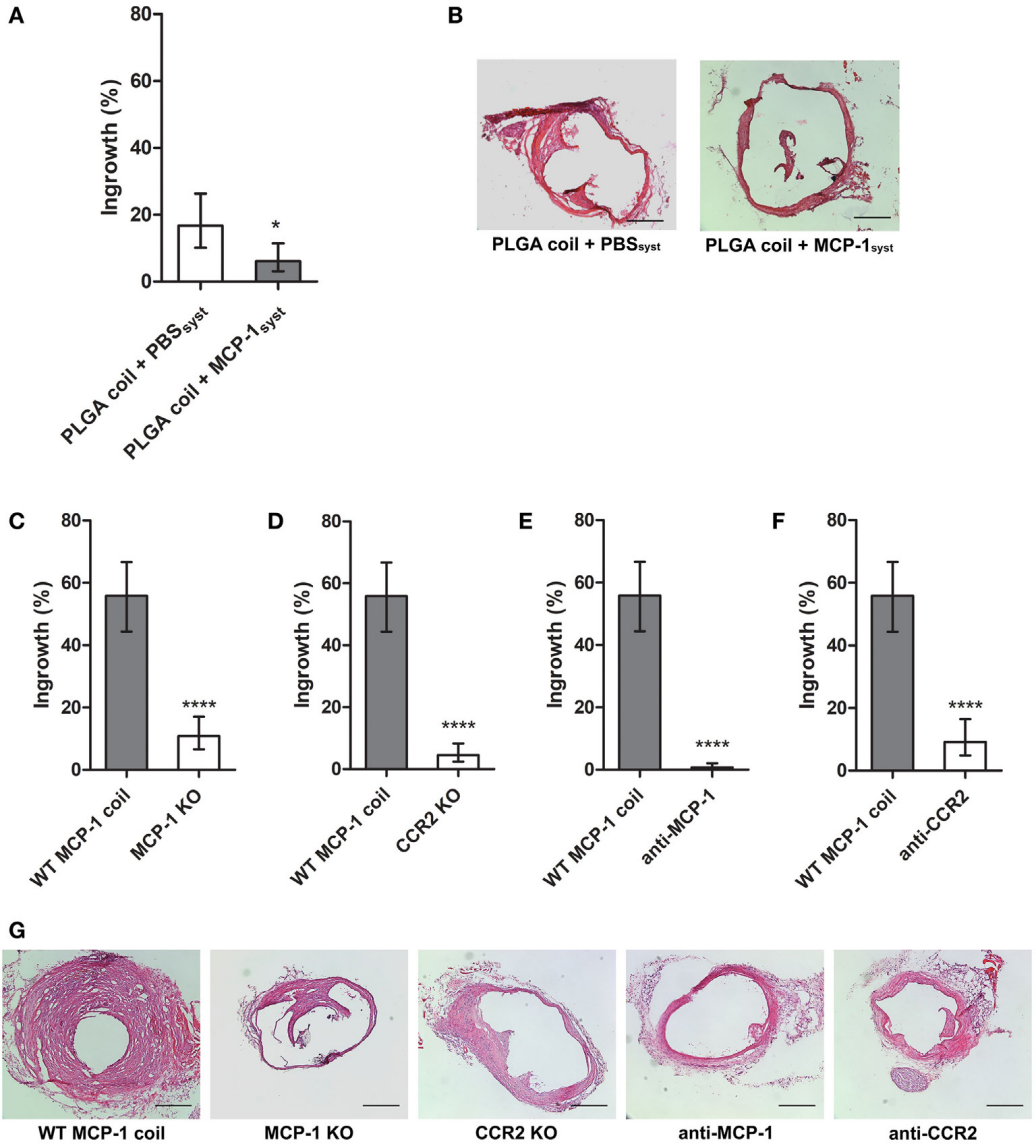
**(A)** Systemic and periodic intraperitoneal monocyte chemotactic protein-1 (MCP-1) administration (*n* = 5) exhibits tissue ingrowth response significantly less than PBS vehicle (*n* = 7). Both groups received IP injections every other day over 3-week coiling period, beginning 2 days prior to control PLGA coil implant. **(B)** Representative H&E images of PLGA coil + PBS_syst_ and PLGA coil + MCP-1_syst_ groups. Aneurysmal ingrowth is significantly decreased in MCP-1 and CCR2-deficient mice. Systemic genetic knockout of **(C)** MCP-1 (*n* = 8) or **(D)** CCR2 (*n* = 6) diminishes ingrowth response versus WT (*n* = 10). Injection of **(E)** anti-MCP-1 neutralizing antibody (*n* = 4) or **(F)** anti-CCR2 selective antagonist (*n* = 5) attenuates ingrowth compared with control (*n* = 10). **(G)** Representative H&E images of experimental groups implanted with MCP-1 eluting coils. Scale bar is 200 µm (*p < 0.05; **p < 0.01; and ****p < 0.0001).

We initially performed a dose response trial of systemic intraperitoneal MCP-1 at doses of 0.1, 1.0, and 10 μg/dose. Animal health and survival rates did not differ by group as a result of systemic MCP-1 treatment, and no difference in ingrowth was detected between dose response groups (data not shown). Thus, 100 µL of 100 µg/mL MCP-1 in PBS was administered every other day over 3 weeks, the same concentration used in solution to create our previously assayed coated coils (14). To verify that the 100 µg/mL dose achieves a systemic therapeutic level, we measured systemic soluble levels of MCP-1 in mice that received systemic MCP-1 versus control PBS. Serum MCP-1 level 6 h post-MCP-1 injection is >4 μg/mL versus vehicle <60 pg/mL (*p* < 0.01, *n* = 5 per group, data not shown). In a separate cohort, we then compared aneurysm tissue ingrowth in mice implanted with poly (lactic-co-glycolic) acid (PLGA) coils and 100 µg/mL systemic MCP-1 versus PBS. Tissue ingrowth with systemic MCP-1 was 5 versus 16% with PBS vehicle (*p* = 0.0144, *n* = 6 and 7, respectively; Figures 1A,B).

#### Cell-Specific Populations

PLGA coil with systemic MCP-1 versus vehicle.

**Figure 2:**
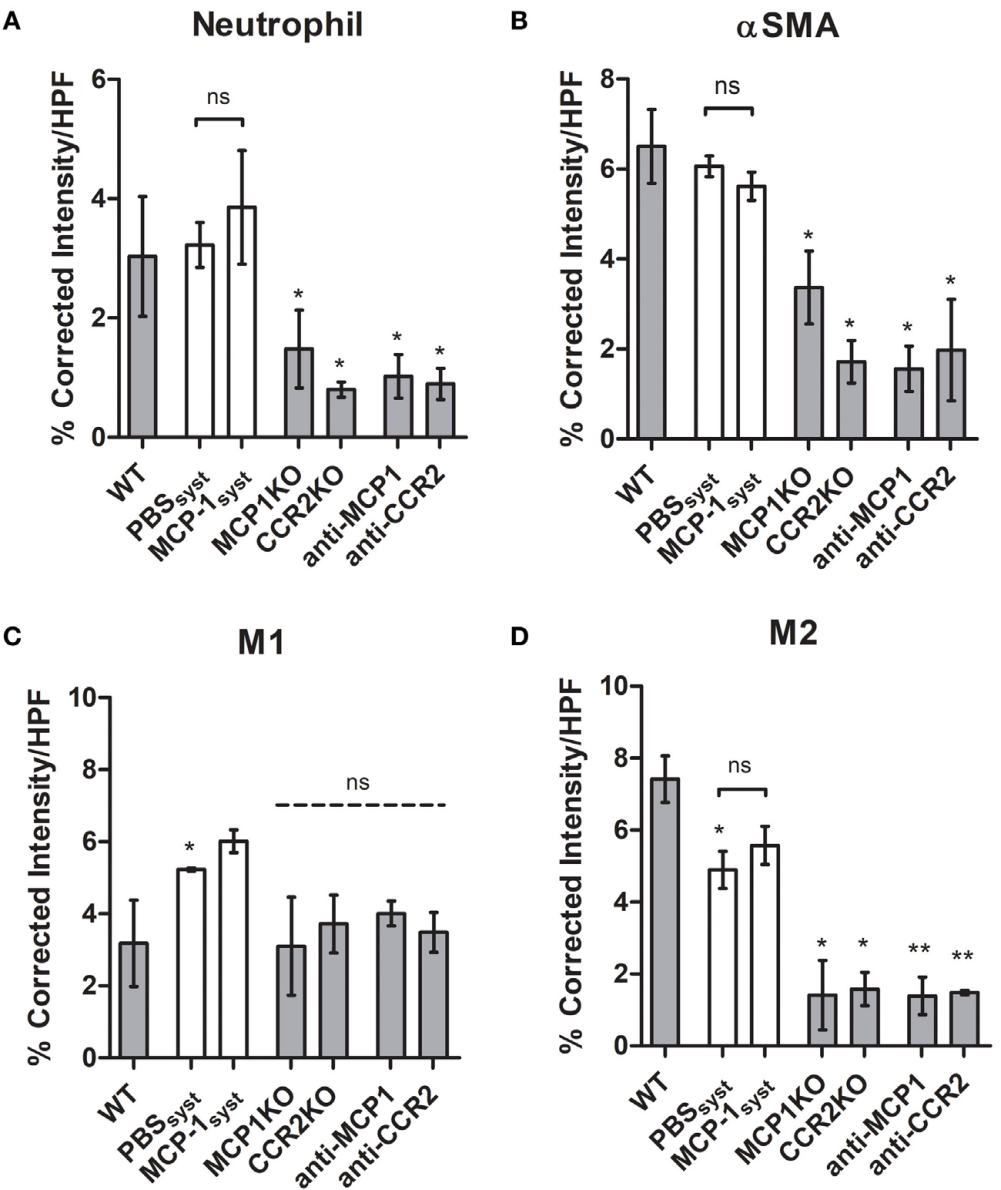
Cell distributions 3-week post-coiling in wild-type, knockout (KO), and systemic blockade cohorts. **(A)** Neutrophil, **(B)** α-smooth muscle actin (αSMA), **(C)** iNOS^+^F4/80^+^, and **(D)** ARG1^+^F4/80^+^. PLGA coils in white, MCP-1 coils in gray. PLGA coil animals were injected with 12 100 μL doses of 100 µg/mL MCP-1 or equal volume PBS, every other day over 3-week coiling period. Abbreviations: MCP-1, monocyte chemotactic protein-1; PLGA, poly(lactic-co-glycolic) acid; HPF, high-powered field.

Three-week post-coiling, no change in neutrophil-stained area was detected between systemically injected MCP-1 (3.9%) or vehicle (3.2%) PLGA coil groups (Figure 2A). PLGA coils with systemic MCP-1 also showed no difference in α-smooth muscle actin (myofibroblast or vSMC)-positive area compared with vehicle (Figures 2A,B). We examined the difference in relative M1 and M2 macrophage populations in aneurysm tissue healing. Compared with PBS vehicle, M1 macrophage-stained area was greater in local MCP-1 coiled treated animals (5.2 versus 6.1%, respectively; Figure 2C). Similarly, M2 macrophage populations in PLGA coiled animals with systemic MCP-1 (5.6%) or vehicle (5.0%) injections were not significantly different (Figure 2D).

### Effect of MCP-1 or CCR2 KO on Aneurysm Healing

Monocyte chemotactic protein-1 eluting coils implanted in murine saccular aneurysm model exhibit increased tissue ingrowth compared with PLGA control, as shown in our previous study (9) and characteristics of aneurysms per group do not differ significantly (Figures S1A,B in Supplementary Material) despite differences in luminal ingrowth (Figure S1C in Supplementary Material). MCP-1 eluting coils were implanted in our murine saccular aneurysm model in MCP-1 KO or CCR2 KO mice to determine the role of MCP-1 or CCR2 depletion on aneurysm tissue healing. There was significantly decreased tissue ingrowth in MCP-1 KO compared with control, expressed as percent cross-sectional area of aneurysm sac: aneurysm tissue ingrowth in MCP-1 KO was 11% versus WT control 56% (*p* < 0.0001, *n* = 8 and 10, respectively; Figure 1C). Furthermore, there was significantly decreased tissue ingrowth in CCR2 KO mice compared with control: aneurysm tissue ingrowth in CCR2 KO was 4.6% versus WT control 56% (*p* < 0.0001, *n* = 6 and 10, respectively; Figure 1D).

### Effect of MCP-1 Antibody or CCR2 Antagonist on Aneurysm Healing

Monocyte chemotactic protein-1 or CCR2 were selectively inhibited to determine their effect on aneurysm tissue healing. Tissue ingrowth significantly decreased with MCP-1 blockade compared with control: tissue ingrowth in animals with MCP-1 eluting coil and anti-MCP-1 was 0.8% versus control 56% (*p* < 0.0001, *n* = 4 and 10, respectively; Figure 1E). Furthermore, there was significantly decreased ingrowth with CCR2 blockade compared with control: ingrowth in animals with MCP-1 eluting coil and CCR2 antagonist is 9.2% versus vehicle 56% (*p* < 0.0001, *n* = 5 and 10, respectively; Figure 1F). Representative H&E stained images of aneurysm ingrowth are depicted (Figure 1G).

#### Cell-Specific Populations

##### MCP-1-eluting Coil with MCP-1 or CCR2 Deficiency

Neutrophil staining was decreased in MCP-1/CCR2 KO (1.5 or 0.8%, respectively) or inhibitor (1.0 or 0.9%) groups compared with WT MCP-1 coil (3.0%). MCP-1/CCR2 KO (3.4 or 1.7%, respectively) or inhibitor (1.6 or 2.0%) groups exhibited a decrease in myofibroblast or vSMC-positive area compared with WT MCP-1 (6.5%) or systemically injected MCP-1 (5.6%) or vehicle (6.1%) PLGA coil groups. WT MCP-1 coiled animal M1-stained area (3.2%) was not significantly different from MCP-1/CCR2 KO (3.1 or 3.7%) or inhibitor (4.0 or 3.5%) groups. M2 macrophages, considered reparative, had increased stained area in the WT MCP-1 coiled animals (7.4%) compared with MCP-1/CCR2 KO (1.4 or 1.6%) or inhibitor (1.4 or 1.5%) groups, which had invariably low M2 macrophage presence.

## Discussion

Taking advantage of commonly recruited immune cell lineages in aneurysms has been suggested as a means to provoke inflammatory tissue-healing (15). Wound healing and vascular repair are marked by inflammatory, degradative and proliferative stages, with eventual M2-like polarization of macrophages to support tissue healing (16). The locally activated tissue upregulates cytokines which perpetuate chronic remodeling until the intraluminal aneurysm sac stabilizes (17). These stages of intracranial aneurysm healing after coiling may be similar to peripheral wound healing, which progresses through hemostasis, inflammation, proliferation of granulation tissue, and resolution (18).

Monocyte chemotactic protein-1 has been investigated in its chemotactic and pro-inflammatory properties, whereas studying its role in vessel healing has been limited to other vascular environments, such as regression of atherosclerotic plaques and systemically inflamed endothelial cell-monocyte adhesion (19, 20). Previous studies using MCP-1 KO mice in diabetic wound healing have shown reduced macrophage recruitment into the local environment (21). We have previously shown that recruitment of this macrophage axis is instrumental in our MCP-1 mediated aneurysm healing model (22). We have also previously shown that MCP-1 contributes to aneurysm healing by means of its downstream mediators (23). Presently, we emphasize the importance of local activity provided by MCP-1 eluting coils.

Using our elastase-induced carotid aneurysm model, we used PLGA coil control with systemic injection of MCP-1 compared with PBS vehicle to allow for a experimentally analogous control to MCP-1 locally eluting coil. Interestingly, the degree of tissue ingrowth in animals implanted with PLGA coil following systemic IP injection of MCP-1 attenuated PLGA coil-induced tissue-healing response in the local aneurysm environment compared with PBS vehicle (Figure 1A). This suggests that, in our hands, a chemotactic effect to the locally inflamed environment is critical for murine aneurysm tissue ingrowth, as administration of systemic MCP-1 may detract from any endogenous local chemotactic gradient to the aneurysm site. Tissue ingrowth is also attenuated by systemic deficiency of both MCP-1 and CCR2 and delivery of systemic MCP-1 neutralizing antibody and CCR2 antagonist, further supporting the mechanistic role of MCP-1 mediated aneurysm healing.

We cross-validate that MCP-1 is critical in the aneurysm tissue-healing pathway. By inhibiting MCP-1 or its receptor CCR2, or by genetic KO of MCP-1 or CCR2, we eliminate the aneurysm tissue-healing response. Both MCP-1 and receptor CCR2 KO mice exhibit decreased tissue ingrowth into the aneurysm lumen (Figures 1C,D). Deficiency of MCP-1 and CCR2 exhibited significant decrease in ingrowth from control, which may be attributable to systemic inhibition of macrophage migration from their bone marrow or circulating monocyte source (9, 24). However, it has been shown that for KO animals, similar or redundant pathways can often be upregulated to compensate from the expected deficient phenotype (24). Therefore, we further cross-validated MCP-1’s critical role in aneurysm healing by studying the effect of selective inhibitors. One caveat is that CCR2 receptor antagonism would affect CCL7, CCL8, CCL11, and CCL13 in addition to baseline MCP-1/CCL2 signaling (25, 26). An additional limitation of our study is the sole use of female animals, as our previous studies and preliminary data exclusively were in females for purposes of bone marrow transplanted chimeras. However, it is possible that gender may have an effect on healing responses and this is a direction for future study in this model. Although MCP-1 inhibitor group was minimally underpowered, the mean difference from control was greater than expected. Application of a systemic MCP-1 neutralizing antibody and CCR2 antagonist also exhibits decreased tissue ingrowth with respect to control (Figures 1E,F).

The induction of inflammatory cell migration is also encouraged by and dependent on local elution of MCP-1 from our coated platinum coils. Specifically, restorative M2-like macrophages and vSMC or myofibroblast differentiation (both visualized with α-smooth muscle actin) are activated in aneurysms treated with MCP-1 eluting coils, whereas this profile is not achieved in systemic MCP-1 administered cohorts (Figures 2B,C). Meanwhile, the cellular phenotype of ingrowth induced by systemic MCP-1 injection does not differ from that of PBS vehicle when using PLGA-coated platinum coils. This enhancement is attenuated in MCP-1/CCR2 KO and blockade groups compared with local MCP-1 administration (Figure 2). M2-like macrophages and myofibroblasts are found to promote aneurysm healing by intrasaccular fibrosis, thereby ameliorating aneurysm rupture (17, 27).

A persistent inflammatory phase of aneurysm wound healing is associated with increased extracellular matrix deposition and progression to fibrotic proliferation (2). Chronic inflammation has also been described as the prodrome of a fibrotic response, as seen in pathogenic processes in other tissues (28). To isolate the aneurysm dome from the parent vessel, ingrowth into the aneurysm should persist beyond the inflammatory and remodeling stages of wound healing such that recanalization does not occur. Therefore, a fibrotic response is desired in aneurysm healing to attenuate re-bleeding events after endovascular coiling.

In conclusion, we find that durable aneurysm healing, as a consequence of inflammatory cell chemotaxis, is dependent on local, and not systemic, administration of MCP-1. The role of MCP-1 is essential for an inflammatory response, which elicits aneurysm healing.

## Author Contributions

SH and KM equally contributed to acquisition and analysis of the data, drafting and revisions of the manuscript. DW, HF, and CJ greatly contributed to data acquisition. SD contributed intellectual content and critical revisions. KH contributed to acquisition and analysis of data and critical revisions to the manuscript. BH contributed to the concept of the work, interpretation of data, and revisions to the manuscript.

## Conflict of Interest Statement

The authors declare that the research was conducted in the absence of any commercial or financial relationships that could be construed as a potential conflict of interest.
