# Supplementary material for: Local Delivery Is Critical for Monocyte Chemotactic Protein-1 Mediated Site-Specific Murine Aneurysm Healing
Source: Front Neurol. 2018 Mar 19;9:158. doi: 10.3389/fneur.2018.00158 (PMC5868072; doi:10.3389/fneur.2018.00158)
Supplement: Supplementary file 1 [file data_sheet_1.PDF]

Supplementary Material

Local Delivery is Critical for MCP-1 Mediated Site-Specific Murine Aneurysm Healing

Siham Hourani, BS[1#], Kartik Motwani, BS[1#*], Daisuke Wajima, MD[1], Hanain Fazal, BS[1], Chad H Jones[2], Sylvain Doré, PhD[3], Koji Hosaka, PhD[1], Brian L Hoh, MD[1]

[1]Department of Neurosurgery; [2]College of Medicine; [3]Department of Anesthesiology, University of Florida, Gainesville, FL, United States

[#]Authors contributed equally to this work

*** Correspondence:** Kartik Motwani, kmotwani@ufl.edu

1 Supplementary Methods

Animals

All animal experiments was performed in accordance with approved protocol #201604771 by the University of Florida Institutional Animal Care and Use Committee and comply with Animal Research: Reporting of *In Vivo* Experiments (ARRIVE) guidelines. Naïve female C57BL/6 (Charles River, Wilmington, MA), MCP-1 KO (004434, Jackson Laboratories, Bar Harbor, ME), or CCR2 KO (004999, Jackson) mice age 6-10 weeks weighing 19-25 g were used in the experiments.

Creation of Coated Coils

MCP-1–coated coils were created using a method previously described [1]. Briefly, standard platinum aneurysm coils (250 µm outer diameter, 2.5 mm length; Cordis, Milpitas, CA) were dipped into an aqueous protein suspension consisting of 100 µg/mL recombinant murine MCP-1 (Sigma-Aldrich, St. Louis, MO) in 50:50 (w/v) poly-DL-lactic glycolic acid (PLGA; Sigma-Aldrich) and dichloromethane anhydrous (Sigma-Aldrich), neutralized with $Mg(OH)_2$ and dried for at least 24 hours at 4°C. PLGA-only coils were created by dipping standard platinum aneurysm coils into aqueous suspension of 1X phosphate buffered saline (PBS; Invitrogen, Carlsbad, CA) in 50:50 PLGA and dichloromethane anhydrous.

Sample Size, Blinding and Randomization

Our power calculations predict that for 80% power to detect specific differences in tissue ingrowth, we will need n=5 per group to detect a mean difference of >30% increased tissue ingrowth, based on preliminary data. When randomized experimental groups resulted in greater numbers of animals than specified, all animals were included in statistical analysis accounting for differences in group size. In all experiments, mice were randomly assigned by cage to each experimental group to minimize between-animal variability per group. Systemic injections were administered via syringes labeled randomly with a numeric code. Surgical procedures and treatments were performed blinded to coil identity or treatment group with treatment modalities stored in blinded containers. Tissue sectioning, de-identification, probing and analyses were performed by separate groups. Digitized histology images were further blinded by random assignment of de-identified samples prior to measurements.

The key to de-identify samples was stored in a password-locked file and unblinded only prior to final data analysis.

Aneurysm Model

Mice were anesthetized with intraperitoneal injections of ketamine (100mg/kg):xylazine (10mg/kg) solution, followed by microsurgical exposure of the right common carotid artery (RCCA) with an operating microscope and adapted from a previously described model [2]. The RCCA was exposed as far proximally and distally as possible (about 1 cm), and a latex cuff was placed around the vessel. The RCCA was next bathed in 10 U/mL porcine pancreatic elastase solution (Worthington Biochemical Corp, Lakewood, NJ) diluted in 1× PBS, for 20 minutes. Vessels were occluded distally and animals returned to normal specific-pathogen-free housing with food and water ad libitum and 12-hour light/dark cycling. After 3 weeks, coils were implanted in carotid saccular aneurysms. Coil implantation was performed by microsurgical insertion into the saccular space, followed by vessel closure using a bipolar electrode. Implanted aneurysms were harvested on day 21 for histologic preparation.

Sustained Systemic MCP-1 Administration with Control PLGA Coil

A dose response trial of recombinant mouse MCP-1 (479-JE/CF, R&D Systems) was performed (n=5 per group): 100uL of 1, 10, 100 µg/mL MCP-1 in PBS was administered intraperitoneally one day prior to PLGA coil implant and then every other day for 3 weeks. There was no difference in ingrowth (data not shown), no neurological symptoms, nor pain or distress among the three doses which would require euthanasia by IACUC Humane Endpoints, so the highest concentration (100uL of 100 µg/mL) was used for the remaining studies in two additional cohorts for MCP-1 and vehicle PBS injection (n=5 and 7, respectively).

Peripheral Soluble MCP-1 Levels following Single Systemic Injection

To verify that mice that received systemic MCP-1 had achieved therapeutic levels of MCP-1, we measured peripheral soluble MCP-1 levels in a separate cohort of mice (n=5 per group) that received systemic MCP-1 (100uL of 100 µg/mL intraperitoneal) versus PBS vehicle, adapted from [3]. Cheek vein serum was collected at a single time point, 6 hours following single MCP-1 or vehicle injection. MCP-1 levels in serum were quantitatively measured using mouse Quantikine MCP-1 ELISA kit (R&D Systems) following manufacturer instructions.

Selective Inhibition of MCP-1 or CCR2

Selective inhibition of MCP-1 or CCR2 was studied by anti-mouse MCP-1 neutralizing antibody (10µg/dose, intraperitoneal; MAB479, R&D Systems) or CCR2 antagonist (0.2mg/dose, intraperitoneal; sc-202525, Santa Cruz, Dallas, TX) by IP injection one day prior to MCP-1 eluting coil implant and then every other day for 3 weeks. Doses were selected for MCP-1 [4] and CCR2 [5] from previous studies.

Histology

After animal perfusion with 4% Paraformaldehyde (PFA; Sigma-Aldrich) in 1x PBS, excised aneurysms were further fixed in 4% PFA overnight, then twice immersed in sucrose baths (18% in 1X PBS at 4°C) overnight. Tissues were embedded in optimum cutting temperature solution (Tissue-Tek, O.C.T. compound, Sakura Finetek USA Inc., Torrance, CA) and frozen over dry ice. Samples were sectioned via cryostat into 5-µm sections from distal aspect of aneurysm, and hematoxylin and eosin staining was performed. Quantitative measurements for aneurysm wall cross sectional area and

intraluminal tissue ingrowth were performed by two blinded observers with Image-Pro Plus software (Meyer Instruments Inc., Houston, TX) following microscopy imaging at 10X on a Olympus IX71 fluorescent scope (Olympus Inc., Center Valley, PA).

Immunofluorescence

Adjacent sections from distal 100µm of fixed-frozen murine carotid aneurysms, as described above, were probed for immunodetection within aneurysm wall and tissue ingrowth, imaged at 20X. The following probes were used to detect cellular presence: myofibroblasts or vascular smooth muscle cells (vSMCs), αSMA (ab5694, Abcam, Cambridge, UK); M1 macrophage, iNOS (PA1-036, Thermo Fisher Sci, Rockford, IL); M2 macrophage, Arginase-1 (ab60176, Abcam); neutrophil, ELANE (ab21595, Abcam), murine macrophage marker, F4/80 (MCA497, Bio-Rad, Hercules, CA). Primary antibodies were incubated overnight at 4°C in the following concentrations: aSMA (1:100), iNOS (1:150), Arginase-1 (1:50), ELANE (1:100), F4/80 (1:100). Murine F4/80 was double-stained to assess M1 and M2 positive cells. All slides were washed (1X TBST, Cell Signaling Technology, Danvers, MA) and incubated with Alexa Fluor (Invitrogen; 488 or 594) antibodies for fluorescent assessment. Vectashield (Vector Laboratories, Burlingame, CA) with DAPI was added for nuclear staining and slide mounting. Control immunonegative slides (data not shown) accompanied each staining round. Relative intensity was calculated using corrected integrated density per field to subtract background staining. Corrected area fluorescence = integrated density – (selected area of interest – mean background fluorescence). ImageJ software (NIH) was used to measure field area and pixel intensities. Percentages represent area staining above non-ingrowth (baseline yield) samples.

Statistical Analysis

We used mixed effect beta regression to compare mean percent ingrowth per tissue section among experimental groups [6]. This model assumes percent ingrowth at section level follows a beta distribution with separate mean parameters for each experimental group. The beta distribution characterizes response variables that are bounded by zero and one (or by 0% and 100%). Because our section-level measurements of % ingrowth were clustered within animal tissue samples (1 sample per animal), we modeled animals as a random effect nested within experimental group. As Beta distribution cannot accommodate absolute zero values, we added a very small increment to zero values prior to fitting our models. We examined residual errors from our fitted model to determine if model assumptions were reasonably met and to identify outliers. We identified a small number of outliers and fitted our regression model both including and excluding the outliers, one each in anti-MCP1 and PLGA+MCP-1_{syst} groups, to determine if they were influential. Within the framework of our fitted models, we estimated mean % ingrowth for each experimental group along with 95% confidence intervals. We performed comparisons of interest between groups using a test based on the t statistic. All model fits and calculations were carried out using SAS Version 9.4 (SAS Institute, Cary, NC). One-way ANOVA with Bonferroni post-hoc test was used for analysis of aneurysm characteristics using Prism 5.0 (GraphPad, San Diego, CA). All means and 95% confidence intervals for cell-specific populations and soluble MCP-1 ELISA were calculated using Prism 5.0.

2. Supplementary Figures

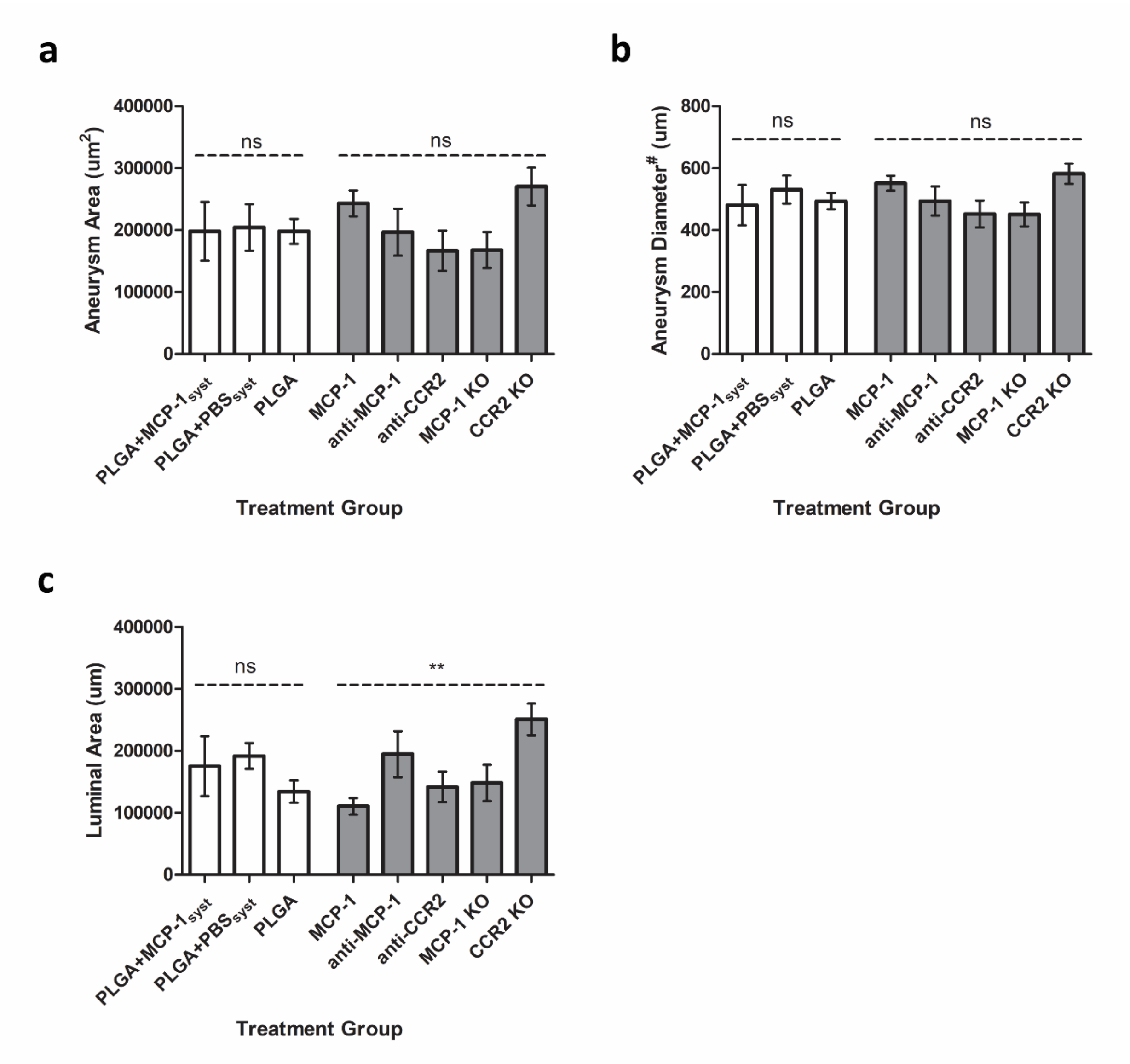

Supplemental Figure 1. Cross sectional area inside vessel wall and lumen were measured in μm^2 for each experimental group to obtain % ingrowth values. Groups with PLGA coil implant are shown in white, and with MCP-1 coil implant are shown in grey. a) Aneurysm cross sectional area does not differ significantly from the respective MCP-1 or PLGA coil control, $p>0.05$ by one-way ANOVA with Bonferroni post-test. b) Average aneurysm diameter for each group does not differ significantly from respective control, $p>0.05$. $^{\#}$Diameter in µm is calculated using vessel cross sectional area, with the geometric assumption that aneurysm wall is approximately circular. Calculated average aneurysm diameter across all groups is 504.6 µm. c) Cross sectional luminal area does not differ significantly from control for PLGA coil groups with systemic injection, $p>0.05$, but is significantly different between MCP-1 coil groups, **$p=0.0031$.

3. Supplementary References

[1] Hoh BL, Hosaka K, Downes DP, Nowicki KW, Fernandez CE, Batich CD, et al. Monocyte chemotactic protein-1 promotes inflammatory vascular repair of murine carotid aneurysms via a macrophage inflammatory protein-1α and macrophage inflammatory protein-2-dependent pathway. Circulation 2011;124(20):2243.

[2] Connolly JES, Winfree CJ, Stern DM, Solomon RA, Pinsky DJ. Procedural and strain-related variables significantly affect outcome in a murine model of focal cerebral ischemia. J Neurosurg 1996;38(3):523-32.

[3] Zisman DA, Kunkel SL, Strieter RM, Tsai WC, Bucknell K, Wilkowski J, et al. MCP-1 protects mice in lethal endotoxemia. J Clin Invest 1997;99(12):2832-6.

[4] Liu J, Jha P, Lyzogubov VV, Tytarenko RG, Bora NS, Bora PS. Relationship between complement membrane attack complex, chemokine (C-C motif) ligand 2 (CCL2) and vascular endothelial growth factor in mouse model of laser-induced choroidal neovascularization. J Biol Chem 2011;286(23):20991-1001.

[5] Furuichi K, Wada T, Iwata Y, Kitagawa K, Kobayashi K, Hashimoto H, et al. CCR2 signaling contributes to ischemia-reperfusion injury in kidney. J Am Soc Nephrol 2003;14(10):2503-15.

[6] Andrew G, Jennifer H. Data Analysis Using Regression and Multilevel/Hierarchical Models. GB: Cambridge University Press - M.U.A; 2007.
